# Pathological Study of Blood Parasites in Rice Field Frogs, *Hoplobatrachus rugulosus* (Wiegmann, 1834)

**DOI:** 10.4061/2011/850568

**Published:** 2011-09-12

**Authors:** Achariya Sailasuta, Jetjun Satetasit, Malinee Chutmongkonkul

**Affiliations:** ^1^Department of Pathology, Faculty of Veterinary Science, Chulalongkorn University, Bangkok 10330, Thailand; ^2^Department of Biology, Faculty of Science, Chulalongkorn University, Bangkok 10330, Thailand

## Abstract

One hundred and forty adult rice field frogs, *Hoplobatrachus rugulosus* (Wiegmann, 1834), were collected in Srakaew province, Thailand. For blood parasite examination, thin blood smears were made and routinely stained with Giemsa. The results showed that 70% of the frogs (98/140) were infected with 5 species of blood parasites, including a *Trypanosoma rotatorium*-like organism, *Trypanosoma chattoni, Hepatozoon* sp. a, *Hepatozoon* sp. b, and *Lankesterella minima*. Pathological examination of the liver, lung, spleen, and kidney of the frogs that were apparently infected with one of these blood parasites were collected and processed by routine histology and subsequently stained with haematoxylin and eosin. Histopathological findings associated with the *Trypanosoma rotatorium*-like organism and *Trypanosoma chattoni*-infected frogs showed no pathological lesions. *Hepatozoon* sp. a and *Hepatozoon* sp. b-infected frogs developed inflammatory lesions predominantly in the liver, demonstrating granuloma-like lesions with *Hepatozoon* sp. meronts at the centre. Tissue sections of *Lankesterella minima*-infected frogs also showed lesions. Liver and spleen showed inflammatory lesions with an accumulation of melanomacrophage centres (MMCs) surrounding the meronts and merozoites. It is suggested that *Hepatozoon* sp. a, *Hepatozoon* sp. b, and *Lankesterella minima*-infections are capable of producing inflammatory lesions in the visceral organs of rice field frogs, and the severity of lesions is tentatively related to levels of parasitemia.

## 1. Introduction

Studies in several geographical regions have indicated anurans infected with variety of blood parasites, including viruses, rickettsiae, several species of protozoans, and microfilariae [1–6]. *Trypanosoma* spp. (Euglenozoa: Kinetoplastida) are heteroxenous and vector transmitted through a variety of mechanisms. The classification of anuran trypanosomes is still in confusion because some species may be polymorphic [7–9]. *Trypanosoma rotatorium* is a polymorphic species with a wide geographical distribution, while *Trypanosoma chattoni* is a monomorphic species that is found in Asia, Europe, and America [7, 10]. *Hepatozoon* spp. (Apicomplexa: Hepatozoidae) are often found in amphibians, appearing as large banana-shaped organisms in the cytoplasm of host erythrocytes. *Hepatozoon* species possess heteroxenous life cycles, with sexual reproduction and sporogony occurring in an arthropod definitive host. Transmission occurs when such an arthropod, infected with mature oocysts, often containing thousands of sporozoites, is consumed by a vertebrate host. Released sporozoites then migrate to the visceral organs, primarily to the liver, and undergo merogony. The meronts are released into the bloodstream where they form gamonts in erythrocytes. *Lankesterella* spp. (Apicomplexa: Lankesterellidae) are recognized as parasites of frogs and some other ectotherms. Merogony and sporogony occur in vascular endothelial cells in the visceral organs of the vertebrate hosts. Mature sporozoites are released into the bloodstream and invade erythrocytes. Intraerythrocytic sporozoites are taken up by vectors during feeding and it is believed that frogs became infected upon the digestion of sporozoite-bearing vectors. Leeches and mosquitoes have been shown to be capable vectors [6]. 

In Thailand, a survey of mainly blood parasites and helminthes of amphibians has been undertaken [10–12]. There are few published data on the pathogenicity of blood parasites infections in amphibian hosts. Experimental infections with *Trypanosoma inopinatum* were lethal to European green frogs and caused haemorrhages, swollen lymph glands, and anaemia [7]. *Trypanosoma rotatorium* can be pathogenic in tadpoles and in heavy infections with trypanosomes accumulate in the kidneys [7]. The rice field frog, *Hoplobatrachus rugulosus *(Wiegmann, 1834), is one of the most widely distributed frogs in this country and is of economic importance as a food source of the local population. In this paper, the blood parasites of naturally infected rice field frogs were examined, and a histopathological study of their visceral organs was undertaken.

## 2. Materials and Methods

One hundred and forty wild rice field frogs, *Hoplobatrachus rugulosus,* from Wang Nam Yen district, Srakeaw province, Thailand were collected from July to October 2007 by a local commercial vendor. The frogs were bled by cardiac puncture, blood was preserved in heparinized tubes and then examined for hematocrit value. A few drops of fresh blood were smeared, fixed with 100% methanol, and stained with Giemsa. Each thin blood smear was examined and classified under a light microscope [5, 9, 13]. The parasitemia was calculated by counting the number of parasites per 5,000 erythrocytes on a thin blood film for evaluating the blood parasite-infected status and recorded.

Visceral organs such as liver, lungs, spleen, and kidney of the frogs that were apparently infected with just one species of blood parasite were then collected and processed by routine histology and subsequently stained with haematoxylin and eosin for histopathological examination. These slides were also observed with a light microscope.

## 3. Results

**Figure 1:**
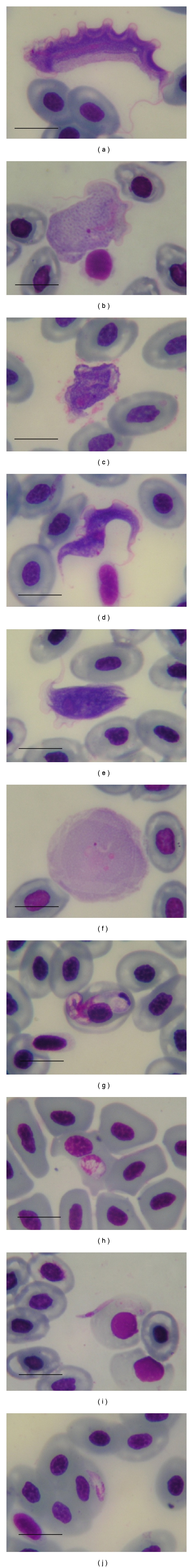
Photomicrographs of the blood parasites of *Hoplobatrachus rugulosus*, *Trypanosoma rotatorium*-like I form (a), *Trypanosoma rotatorium*-like II form (b), *Trypanosoma rotatorium*-like III form (c), *Trypanosoma rotatorium*-like IV form (d), *Trypanosoma rotatorium*-like V form (e), *Trypanosoma chattoni* (f), mature gamont of *Hepatozoon* sp. a in red blood cell associated with large parasitophorous vacuole (g), mature gamont of *Hepatozoon* sp. b in red blood cell (h), extracellular sporozoite of *Lankesterella minima* (i), merozoite of *Lankesterella minima* in red blood cell (j) Giemsa's stain, (Bar = 10 *μ*m).

**Figure 2:**
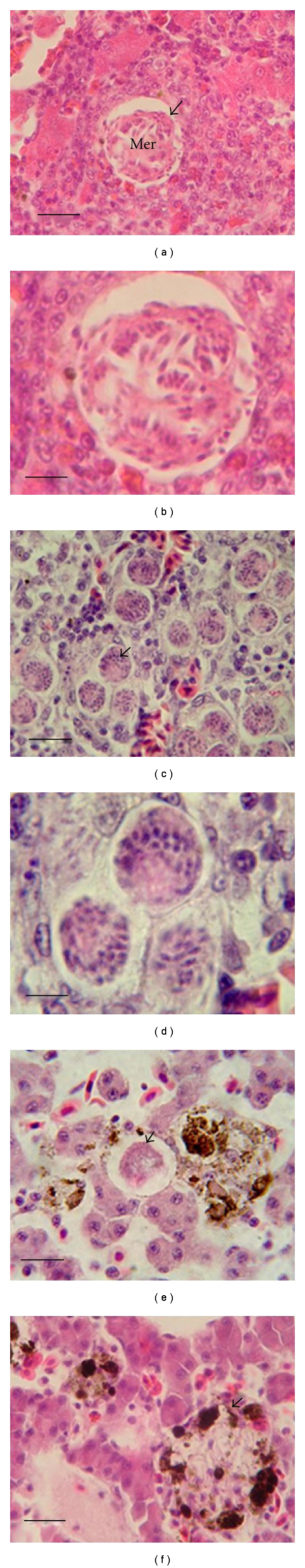
Histopathology findings of the liver tissue of frogs infected with parasites *Hepatozoon* sp. a, *Hepatozoon* sp. b, and *Lankesterella minima*. Meronts (arrowhead in (a), and enlarged version in (b) of *Hepatozoon *sp. a in liver with evidence of lymphocyte and eosinophil infiltration resembling a granuloma-like lesion (a-b), diffuse multifocal meronts of *Hepatozoon *sp. b with infiltration by a few inflammatory cells (c-d), meront (arrowhead in (e)) of *Lankesterella minima *presented in liver, with an increase in melanomacrophages centre (MMCs) and a mild tissue reaction (e), evidence of liver damage associated with a merozoite of *Lankesterella minima* surrounded by an MMC (f), Mer = meront, haematoxylin-eosin (a, c: Bar = 20 *μ*m and b, d: Bar = 6 *μ*m and e, f: Bar = 15 *μ*m).

Of naturally infected frogs, 70% (98/140) were positive for blood parasites. Hematocrit values were not statistically different between the infected (*n* = 98) and noninfected frogs (*n* = 42) at 22.64 ± 5.31 and 22.81 ± 5.82 (mean ± SD), respectively, (*t* = 0.167). The parasites were identified as 5 species: a* Trypanosoma rotatorium*-like organism (I–V forms), *Trypanosoma chattoni*, *Hepatozoon* sp. a, *Hepatozoon* sp. b, and *Lankesterella minima* (Figures 1(a)–1(j)). Prevalences of frogs apparently infected with one species of the blood parasite only were: the *Trypanosoma rotatorium*-like organism (11.4%, 16/140), *Trypanosoma chattoni* (22.1%, 31/140), *Hepatozoon* sp. a (4.3%, 6/140); *Hepatozoon* sp. b (1.4%, 2/140), and *Lankesterella minima *(7.1%, 10/140) respectively (Table 1). Histopathological study of the visceral organs in the case of the *Trypanosoma rotatorium*-like organism and *Trypanosoma chattoni*-infected frogs with average parasitemias of 0.01% of erythrocytes, showed no pathologic lesions in the tissues. There was no detectable macroscopic change in the various organs. *Hepatozoon* sp. a- and *Hepatozoon* sp. b- infected frogs, with parasitemias of 0.28% and 0.49% of erythrocytes, respectively, showed pathologic lesions predominantly in liver. The lesions revealed subacute to chronic inflammation. There were evidence of lymphocyte and eosinophil infiltration around the meronts of *Hepatozoons* sp. a and *Hepatozoons* sp. b as granulomatous-like lesions. However, in infected frogs with average parasitemias of 0.01% of erythrocytes, there were no remarkable lesions. The frogs that were infected with *Lankesterella minima, *with average parasitemias of 0.15% of erythrocytes, showed pathologic lesions at the meront and merozoite stages in the various visceral organs, liver, lung, spleen, and kidney. It was found that the meront and merozoite stages were surrounded by the melanomacrophage centers (MMCs). The lung and kidney with merozoite stages also showed tissue damage with a mild degree inflammation. However, in infected frogs with average parasitemias below 0.01% of erythrocytes, no remarkable lesions could be recognized (Figures 2(a)–2(f) and Table 1).

## 4. Discussion and Conclusion

Blood parasites that were observed in wild rice field frogs, *Hoplobatrachus rugulosus* in Wang Nam Yen district, Srakaew province, Thailand from July to October 2007, included trypanosomes, haemogregarines, and lankesterellids, with trypanosomes having highest prevalence. The morphologies and measurements of the *Trypanosoma rotatorium-*like species (I–V forms) and *Trypanosoma chattoni* observed in this study are similar to the observation of Werner [5]. The *Hepatozoon* was differentiated into species a and b by the characteristics of the parasitophorus vacuole [13]. However, the species could not be identified with any currently known to science. For the *Lankesterella*, sporozoites observed in the rice field frog were similar in size and morphology to those of *Lankesterella minima*, which is the most prevalent species reported from frogs in the USA, Europe, and Asia [4, 11, 12]. Thus, the *Lankesterella* in this report could be identified as *Lankesterella minima*. 

There was no statistical difference between the hematocrit value of infected and noninfected frogs. That could be because the blood parasites form part of the normal fauna of wild frogs. All the frogs were clinically normal and, thus, considered suitable for human consumption. In the case of the trypanosomes, including the *Trypanosoma rotatorium*-like organism and *Trypanosoma chattoni*, there was no evidence of pathological lesions in any visceral organs. It is suggested that the number of blood parasites found in those frogs with average parasitemias at or below 0.01% of erythrocytes did not affect frog health or produce any clinical signs. *Hepatozoon *sp. a and b meronts and *Lankesterella minima* meronts and merozoites produced pathological lesions predominantly in the liver with subacute to chronic inflammation [14, 15]. Those lesions resembled granulomas in liver which corresponded to cell-mediated immune response [15, 16]. The meronts and merozoites of *Lankesterella minima *presented in various organs, that is, liver, lung, and kidney, producing mild degree inflammatory response [15]. The stages developed in the reticuloendothelial system, associated with the melanomacrophage centers (MMCs) [9]. In the case of haemogregarine and lankesterellid infections, the severity of histopathological lesions observed appeared to be tentatively related to the levels of parasitemia.

From the results obtained, it can be concluded that wild rice field frogs,* Hoplobatrachus rugulosus*, were infected with 5 species of blood parasites, including 2 species of trypanosomes, 2 of *Hepatozoon,* and one lankesterellid. Frogs apparently with just one of these infections showed pathological lesions in the visceral organs only in the case of *Hepatozoon* sp. a and b (meronts mainly in liver) and *Lankesterella minima *(meronts and merozoites in liver, lung, spleen, and kidney). No evidence of any inflammatory lesion was found in the various organs in the case of the *Trypanosoma rotatorium*-like organism and *Trypanosoma chattoni. *The severity of the lesions in the wild frogs infected with blood parasites ranged from severe to mild in the order *Hepatozoon* sp. a, *Hepatozoon* sp. b, *Lankesterella minima*, the *Trypanosoma rotatorium*- like organism and *Trypanosoma chattoni, *respectively. In addition, the severity of the pathological lesions appeared to relate to the levels of parasitemia (percentages of erythrocytes infected). However, the infected frogs did not show any clinical signs of sickness although blood parasites may be stress factors including immunosuppressive conditions that render hosts more susceptible to secondary infection. This information will be useful for health monitoring in the frog management industry and ecological conservation.

## Figures and Tables

**Table 1:** The average parasitemias of the infected frogs that were apparently infected with just one species of blood parasite.

Blood parasite	Prevalence (%) (+ve/total frogs)	Average parasitemias (percentages of erythrocyte infected, mean ± SD)
*Trypanosoma rotatorium*-like organism	11.4% (16/140)	0.01 ± 0.01
*Trypanosoma chattoni*	22.2% (31/140)	0.01 ± 0.01
*Hepatozoon *sp. a	4.3% (6/140)	0.12 ± 0.18
*Hepatozoon *sp. b	1.4% (2/140)	0.12 ± 0.20
*Lankesterella minima*	7.1% (10/140)	0.15 ± 0.45
